# The role of health and advocacy organisations in assisting female sex workers to gain access to health care in South Africa

**DOI:** 10.1186/s12913-019-4552-9

**Published:** 2019-10-24

**Authors:** Nosipho Faith Makhakhe, Anna Meyer-Weitz, Helen Struthers, James McIntyre

**Affiliations:** 1 School of Applied Human Sciences, Department of Psychology, University of KwaZulu-Natal, Howard College Campus, Memorial Tower Building, 238 Mazisi Kunene Road Glenwood, Durban, 4041 South Africa; 2 Anova Health Institute, 12 Sherborne Road, Parktown, Johannesburg, 2193 South Africa; 3 Anova Health Institute, Honorary research associate in the Division of Infectious Diseases & HIV Medicine, Department of Medicine, University of Cape Town, Cape Town, South Africa; 4 Anova Health Institute, Honorary professor in the School of Public Health & Family Medicine, University of Cape Town, Cape Town, South Africa

**Keywords:** HIV, Health care, Stigma, Discrimination, Female sex workers, Health and advocacy organisations

## Abstract

**Background:**

Globally, female sex workers (FSWs) are considered a key population group due to the high HIV prevalence. Studies show that there are various factors in some contexts that render FSWs marginalised, which limits their access to sexual reproductive health (SRH) services. Access to SRH services are particularly challenging in countries where sex work is criminalised such as is the case in South Africa. Evidently, there are alternative ways in which FSWs in this context receive non-stigmatising SRH care through non-governmental organisations. The aim of this study was to understand the functioning of these non-governmental health care services as well as to document the experiences of FSWs utilising these services.

**Methods:**

Eleven focus group discussions were held with 91 FSWs. In addition, 21 in-depth individual interviews with researchers, stakeholders and FSWs were conducted. Interview guides were utilised for data collection. Informed consent was obtained from all participants. Data were analysed thematically.

**Results:**

The FSWs expressed challenges related to SRH care access at public health facilities. The majority felt that they could not consult for SRH-related services because of stigma. The non-governmental health and advocacy organisations providing SRH services to FSWs through their mobile facilities utilising the peer approach, have done so in a way that promotes trust between FSWs and mobile health care providers. FSWs have access to tailored services, prevention materials as well as health information. This has resulted in the normalising of HIV testing as well as SRH seeking behaviours.

**Conclusion:**

This study has established that health and advocacy organisations have attempted to fill the gap in responding to SRH care needs of FSWs amidst intersecting vulnerabilities. FSWs’ engagement with these organisations has encouraged their willingness to test for HIV. However, it is important to note that these organisations operate in urban areas, thus FSWs operating outside these areas are most likely exposed to compounding health risks and lack access to tailored services.

## Background

Female sex workers are generally regarded as a key population group because they are at higher risk of contracting and transmitting HIV. In low- and middle-income countries, FSWs are 13.5 times more likely to be HIV positive than women in the general population [1]. Evidently, nearly 40% of FSWs in sub-Saharan Africa are living with HIV [2]. This is due to the nature of their work which involves multiple partnerships, limited access to health care because of stigma and discrimination, as well as criminalisation [3–6]. Studies show that FSWs are knowledgeable about HIV and safe sex practices but struggle to negotiate condom use, especially when faced with challenging situations such as clients offering more money for unprotected sex [4, 7–14]. An integrated biobehavioural survey (IBBS) conducted in three South African cities among FSWs showed an HIV prevalence of 71.8% in Johannesburg, 39.7% in Cape Town and 53.5% in Durban [15]. A call has been made for concerted efforts to address the susceptibility of FSWs to new HIV infections if South Africa is to achieve the UNAIDS 90–90-90 targets. These targets stipulate that 90% of all people need to know their status, 90% of all people with HIV should be on antiretroviral therapy (ART) and 90% of those receiving ART to achieve viral suppression by 2020 [7].

Although FSWs do receive HIV prevention materials such as condoms, little is known with regard to the number of FSWs accessing ART, as well as those being retained in the treatment continuum [16–18]. Sex workers are in need of regular targeted health care interventions to effectively respond to HIV, this will curb the spread of new infections, of which 20% was said to be related to sex work in 2010 [2, 19–21]. The response to provide FSWs with access to better health care in South Africa has been through non-governmental organisations who have SRH and advocacy programmes targeting sex workers. Some of these organisations provide services ranging from prevention interventions, testing, screening and treatment for sexually transmitted infections (STIs). Since the completion of this research, these organisations have expanded their services to include the initiation of ART and pre-exposure prophylaxis (PrEP). However, little has been reported with regard to FSWs’ experiences with these organisations and the influence they have on FSWs’ health seeking behaviour, as well as the ways in which these organisations operate, particularly their approach in engaging with this key population. The aim of this study was to understand the functioning of these health care services as well as to document the experiences of FSWs in utilising these services.

## Methods

### Study setting

This study was conducted as part of the formative assessment prior to the IBBS that took place in 2013 and 2014 among inner-city FSWs in three South African cities: Durban, Johannesburg and Cape Town [15].

### Research design

Qualitative methods were used to explore and understand the context in which FSWs live and work. These included focus group discussions (FGDs) and key informant interviews (KIIs). This study was based on a social constructivist paradigm which suggests that individuals experience multiple constructed realities in their social world [22]. The social constructivist paradigm in qualitative research views the relationship that the researcher has with the participant as subjective, whereby the participant is viewed as the expert in his or her own life due to their own unique experiences [22]. We were able to engage FSWs as individuals and as a group with the purpose of attempting to understand both their personal and collective experiences regarding their access to health care in South Africa.

### Sampling technique

The selection of the initial contacts was guided by the national coordinator of a sex work organisation located in Johannesburg. This national coordinator was purposefully sampled and participated in the study as one of the key informants, and then connected us with provincial coordinators from each city. Each provincial coordinator who was interviewed as a key informant connected us to FSW peer educators in each province who then also formed part of the FGDs and KIIs. Furthermore, the peer educators assisted us with a chain referral of FSWs to participate in the FGDs. Other FSWs were snowballed into the study.

We purposefully sampled researchers and stakeholders from health and advocacy organisations who focus on conducting research as well as providing health care and psychosocial services to sex workers.

### Sample

A total of 11 FGDs were conducted with 91 FSWs. These included four FGDs in Durban (39 participants in total), three FGDs in Johannesburg (27 participants in total), and four FGDs in Cape Town (25 participants in total) (see Table 1). A total of 21 KIIs were conducted, five in Durban (three FSWs, one researcher and one stakeholder), nine in Johannesburg (four FSWs, three researchers and two stakeholders), and seven in Cape Town (five FSWs, one researcher and one stakeholder). Because seven FSWs participated in both an FGD and a KII, the total sample size of FSWs was 96.

**Table 1:** Demographic information of 96 female sex workers participating in the focus groups discussions and key informant interviews in South Africa

	Durban		Johannesburg		Cape Town	
	n	%	n	%	n	%
Total	39	100%	31	100%	26	100%
Age group (years)						
19–23	9	23%	2	6%	1	4%
24–28	12	31%	4	13%	4	15%
29–33	7	18%	11	35%	5	19%
34–38	5	13%	7	23%	8	31%
39+	6	15%	7	23%	8	31%
Educational level						
No school	2	5%	0	0%	0	0%
Primary school	2	5%	0	0%	0	0%
Secondary school	27	69%	22	71%	22	85%
Matric (Grade 12)	6	15%	8	26%	3	11%
Tertiary	2	5%	1	3%	1	4%
Language						
IsiZulu	28	72%	10	32%	1	4%
IsiXhosa	4	10%	12	39%	18	69%
Sesotho	4	10%	5	16%	1	3%
Other	3	8%	5	16%	6	23%
Race						
Black	37	95%	31	100%	21	81%
Colored	2	5%	0	0%	5	19%
Indian	0	0%	0	0%	0	0%
White	0	0%	0	0%	0	0%
Country of origin						
South Africa	38	97%	24	77%	25	96%
Zimbabwe	0	0%	6	19%	0	0%
Lesotho	1	3%	1	3%	0	0%
Zambia	0	0%	0	0%	1	4%

### Data collection and procedure

The FGDs and KIIs were conducted by two research assistants, each with a university education; while one facilitated, the other took notes. We conducted both FGDs and KIIs because FGDs elicit information that is generally known by the participants, and at times most group members tend to agree with the perspectives held by the majority. On the other hand, KIIs are conducted with various experts and experienced members of the population under study [23]. As researchers we also felt that through KIIs we could probe into sensitive issues that could be hard for participants to answer in a group, as well as to get more in-depth information that could have been missed in the FGDs; thus, employing both methods as a way of triangulation made up for the limitations of each data collection method [23]. As researchers we felt that using both methods would strengthen the depth of our findings.

Data collection in the three cities took place at venues recommended by the FSWs. KIIs were conducted in a private room with the participant and the researchers to ensure privacy. Two semi-structured interview guides were used to collect data (interview guides attached as Additional files 1 and 2). One guide was for FGDs. This guide had two main sections: The first section covered demographic information; origin of the FSWs in the three cities; the different types of sex work; where and when they work; the type of clients; challenges they face in doing sex work and health care access. The second section focused on the feasibility of conducting an IBBS. The second guide was for KIIs which covered similar discussion topics. We used the same KII guide for FSW peer educators, researchers and stakeholders.

Interviews were first conducted with the national and provincial coordinators of the sex work organisation, as well as researchers and stakeholders, to provide a general understanding of the sex worker community. These were followed by FGDs and KIIs with FSW peer educators and general FSWs. As researchers, we felt that there was some information coming out from the FGDs that we wanted to explore further with the key informants. The key informants were made up of peer educators who were current and former sex workers, as well as general FSWs who provided insights on some issues that needed further clarification. The peer educators also worked closely with the support organisations and gave detailed accounts on the role of these organisations from both a provider and user perspective.

All interviews were conducted in the participants’ preferred languages which included isiXhosa, isiZulu, Sesotho and English. The interviews lasted approximately 45 min for KIIs and 120 min for FGDs. The sessions were also recorded with the participants’ consent. Data were collected between January and February 2013.

### Data analysis

The first author, who was also one of the research assistants, translated and transcribed the data verbatim from IsiXhosa, isiZulu and Sesotho to English. These translated transcripts were further reviewed by the other research assistant who is proficient in the four languages. He listened to the interview recordings and reviewed the translated and transcribed transcripts and also assisted with the data analysis to ensure data quality and rigour. Data were analysed using a thematic approach. The authors coded the translated transcripts, and after extensive consideration of the coded transcripts, the authors reached a consensus about the emerging themes. To ensure analytical rigour, data management, and cross focus group analysis, data were analysed using Microsoft Excel software program. Each Excel spreadsheet consisted of a particular theme and related sub-themes. These were populated with various quotations related to that theme and sub-theme as communicated by participants in a focus group. Each quotation was marked with a focus group number and each participant was given a unique identifier. This enabled us to see what different participants were saying concerning a particular theme across different focus groups in each city. A similar technique was used for each KII. This method of analysis was taken from the six-step process as outlined by Braun and Clarke [24] to translate and transcribe the data, familiarise oneself with the data by reading and rereading, generate codes from the data, generate themes from the codes, define and refine the identified themes, and employ the identified themes in the final presentation of the study findings.

### Ethical considerations

All participants provided written informed consent to participate in the study and have their quotes utilised provided that their identities are kept confidential. Participants were also encouraged to use pseudonyms for anonymity. During the FGDs, the participants were asked not to share the contents of the discussions with anyone outside of the group to encourage confidentiality. Ethical permission was obtained from the Ethical Review Board of the University of Cape Town, South Africa (IRB number 00001938).

## Results

The majority of participants were street-based black isiXhosa and isiZulu speaking South African FSWs between the ages of 19 and 60 years. Foreign FSWs were from Zimbabwe, Zambia and Lesotho. The education attainment of most FSWs was up to secondary level schooling, which the majority did not complete. Two participants had no schooling. Very few FSW participants had undergone tertiary education (See Table 1).

Using a thematic analysis, five broad themes were identified for aspects related to health care access, namely, barriers to health care, health and advocacy organisations, the role of health and advocacy organisations, benefits of health and advocacy organisations, and challenges experienced by health and advocacy organisations working with FSWs.

### Barriers to health care

According to the FSWs in this study, some health care providers in the public health sector make it difficult to access services because of their unwillingness to engage with FSWs as patients in a neutral non-discriminatory manner. They allow their own moral judgements to interfere with their primary role as service providers, and fail to engage with the sexual risk factors that FSWs face. Furthermore, the participants felt that it is difficult going to public facilities when presenting with sexually transmitted diseases because of a non-supportive environment.

*“In clinics we get stigmatised, you go to a nurse and when you tell her that a condom burst during sex and you tell her that you are a sex worker, the nurse will call her colleagues and they will ask you why you selling sex and you so young? And then continue to make fun of you” (FGD4, FSW, Durban).*

*“It’s easy for me to go to the clinic when I am coughing, and my chest is paining, or my stomach is sore, but when it comes to sexual diseases you need to speak the truth, but you find that you can’t speak the truth because of the attitude of nurses” (KII4, FSW peer educator, Cape Town).*

The public health sector is also known to be overburdened and the system has operational challenges such as a lack of adequate medical infrastructure, shortage of medical personnel, and overworked staff. Thus, some FSWs have reported going to these facilities and not receiving the needed help. The long waiting times also have financial implications for FSWs due to the opportunity cost of spending long hours at the health facilities instead of being at work. In order to avoid these challenges some FSWs opt for private consultations; however, not all FSWs are able to afford a private doctor due to the high consultation fees and medical costs, thus the majority rely on an ineffective public system.

*“Some FSWs prefer to go to private doctors but a doctor will not give you equal attention. They say they prefer private doctors because there are long waiting times at the clinic” (FGD1, FSW, Durban).*

*“When you go to this organisation, they will refer you to hospital for a pap smear but they (the hospital) will keep postponing your date of when you should come, until you end up not being able to go, so we don’t get proper care” (FGD3, FSW, Cape Town).*

### Health and advocacy organisations

There are various non-governmental organisations who, with the aid of donor funding, provide SRH services to FSWs in the three cities. The FSWs in Durban mentioned Lifeline, TB/HIV Care Association (THCA), and Sisonke, which focuses mainly on advocacy. Those in Johannesburg mentioned Wits Reproductive Health and HIV Institute (Wits RHI) who work with a sex worker-friendly clinic, and Sisonke. The Cape Town FSWs mentioned the Sex Worker Advocacy Taskforce (SWEAT), Sisonke, THCA and the Desmond Tutu HIV Foundation.

*“Lifeline and TB/HIV Care provide services for FSWs” (FGD4, FSW, Durban).*

*“The support organisations are Sisonke and Wits RHI, all sex workers come to the clinic, even those coming as far as the East Rand because they are well taken care of in this clinic, it is especially for sex workers” (KII7, FSW, Johannesburg).*

*“Desmond Tutu foundation was the first organisation which helped us with HIV testing and now we have TB/HIV Care and SWEAT” (KII1, FSW, Cape Town).*

However, it is important to note that these organisations operate in urban areas and are accessed by FSWs trading in and around the major cities. Other groups of FSWs, with intersecting vulnerabilities such as FSWs who operate in poor rural settings, do not have access to alternative health care services.

### The role of health and advocacy organisations

The organisations mentioned above provide mobile facilities that go out daily to deliver health care services to FSWs. These outreach services facilitated by peer educators operate at different times of the day so as to reach out to FSWs working in different contexts such as on the streets and at brothels. The provision of these services is a response to the need for non-discriminatory SRH care needed by FSWs; these services are provided at no cost to FSWs. Due to their tailored response, these services function at convenient times and are cognisant of FSWs’ time constraints and thus FSWs are not kept waiting and they can access health care on the street during their working time or during their less busy hours.

The types of health services provided focus mainly on SRH services such as HIV testing and screening for other sexually transmitted infections (STI), with some also providing tuberculosis (TB) screening. The sex worker-sensitive clinic in Johannesburg provides a comprehensive service that includes antiretroviral (ARV) treatment as well as PrEP which is also provided by the THCA working in Durban and Cape Town.

*“Basically, we provide health services for sex workers here in the clinic and we also do outreach. We provide services from 08:00 to 11:00 and then the team goes out to brothels from 11:00 to 17:00. Then, on Fridays we target street based FSWs from 11:00 to 15:00. We provide family planning, Pap smear screening, HIV, STI and TB screening and ARVs, as well as treat for minor ailments. We also provide psychosocial support in the form of a creative space where sex workers can offload and debrief about their troubles” (KII4, researcher, Johannesburg).*

*“We have a five-year CDC [Centers for Disease Control and Prevention] funded grant to do HIV prevention in sex workers among male, female, and transgender in the Cape Town metro and eThekwini municipality. We have been asked in these five years to reach 80% of sex workers with behavioural change interventions and HCT [HIV Counselling and Testing] and those who are positive 90% should be linked to care” (KII3, researcher, Cape Town).*

SWEAT and Sisonke in all three provinces are advocacy organisations aimed at providing awareness to government structures regarding the decriminalisation of sex work and its benefits, particulary the protection of FSWs’ human rights. Both SWEAT and Sisonke provide peer education which is carried out by current and former sex workers, as well as condom distribution and psychosocial support in the form of a creative space. This creative space affords sex workers, both male and female, opportunities to come together to support each other and speak about their personal experiences and the challenges they face in the sex trade.

Due to their limited service provision at the time of this study, some of these organisations used to refer FSWs to public hospitals and clinics for further ongoing care. They also went as far as to provide training to public health care providers to be sensitive to the needs of FSWs and promote a non-discriminatory environment:

*“As a support organisation we have partners in clinics that we use that know that this one is a sex worker. So, if one has a discharge, they will ask what happened, but they won’t ask why. We use a drama group to educate them about sex workers, to say there are people out there who are like this, they exist and are female and they do this work” (KII4, FSW peer educator, Cape Town).*

However, as mentioned by the participants, the referral letters coming from support organisations were not always well received due to the internalised stigma felt by some FSWs because the organisations are known to primarily target them. In some instances, FSWs felt that producing the refferal letter to a public health care provider potentially resulted in a loss of privacy because it revealed a person’s sex work identity against their will; thus, FSWs felt exposed, with some preferring to be accompanied by a representative of the support organisation as a protective factor. Preoccupation with work was another challenge that made referrals difficult because some FSWs resisted attending to certain STIs associated with the increase of ‘vaginal tightness’ for client satisfaction.

*“We get referred to clinics such as clinic X, and C. When the nurses see that I have a referral letter from this organisation they tend to say funny things. Personally, I don’t go to those clinics on my own. I think it’s better if I go with someone from the support organisation” (FGD4, FSW, Durban).*

*“We do get referred at the clinics but some of us don’t follow up. There are those, when they get an STI that looks like cauliflower, they ignore that one, because it makes the client happy because it makes the vagina hot and tight. As much as they are sick, they always work, and their life comes after” (FGD1, FSW, Durban).*

A researcher from one of the health organisations at the time of this study expressed a sense of frustration that there were still some FSWs who resisted follow-up care for HIV management. This could be due to denial and fear of stigma to a point where a patient may opt to face the possibility of death rather than to accept that they are HIV positive and are in need of ARV treatment.

*“We test for CD4 count and we find someone with a CD4 count of 35 and we track them, call them and tell them that we will take them for further care and they refuse, you then cannot force someone to access care. They would rather die, and we cannot handcuff them. You can call an ambulance but it’s hard when people don’t want to go” (KII3, researcher, Cape Town).*

### Benefits of health and advocacy organisations

Some FSWs expressed satisfaction and a sense of agency that they cultivated during their engagement with health care providers from these support organisations. They felt that they could engage with these health care providers openly and truthfully without fear of judgement. Receiving accurate knowledge in a dignifying manner was empowering and it fostered in some FSWs a need to engage in positive health seeking behaviours which resulted in some becoming peer educators.

*“I came to this organisation that was before I became a peer educator. Since I’ve been a peer educator, I have never contracted anything because I got taught how to protect myself and use condoms” (KII9, FSW, Johannesburg).*

The visibility and accessibility of mobile health care facilities has created a positive culture of normalising regular HIV testing among FSWs; this has lessened the stigma associated with HIV testing among this group. This health seeking practice, when nurtured, can encourage FSWs to make better choices regarding their health, especially when it comes to safe sexual practices.

*“Women do not have a problem with testing for HIV because they test regularly when Wits RHI does outreach and provide testing” (FGD1, Johannesburg).*

The positive views expressed by FSWs concerning these organisations is further corroborated by one of the programme managers working closely with FSWs. This programme manager asserted that their organisational approach was based on the building of trust with this vulnerable population, ensuring that the health care staff on the ground is sensitive to the needs of FSWs through open communication and patient confidentiality. The provision of a comprehensive one-stop health facility has made service provision easy and convenient for FSWs which has increased the number of FSWs accessing the services.

*“We have managed to build a trustworthy relationship with sex workers in a way that they see this organisation as their home. They are very comfortable to come to us and share their deepest secrets with our team. We have managed to gain their trust. The fact that we provide them with a comprehensive service has encouraged them to test and getting ARVs from our programme has encouraged them to test more often. They are not shy to ask for condoms, they have a very strong bond with the team” (KII4, researcher, Johannesburg).*

### Challenges experienced by health and advocacy organisations working with FSWs

There are various structural and interpersonal challenges that organisations experience because of working with FSWs. These challenges involve FSWs who are under the influence of drugs or alcohol not being able to get tested because of intoxication, as well as mobile units being identified as providing services to FSWs, and the police using that knowledge to follow and track the FSWs. Due to criminalisation of sex work the police incarcerate FSWs for loitering or carrying condoms. The police are also known to violently harass the FSWs which infringes on their human rights.

*“There are some challenges that we are having; the more our mobile unit becomes visible even though it’s not branded as a sex worker clinic, we have had instances where the police have been following women that come to our mobile units and later arresting them, not instantly but later. There has been some kind of profiling taking place that you are a sex worker if you go into this thing (mobile clinic). Another challenge is that FSWs are on drugs and alcohol, when they come to test, we cannot test them because they are intoxicated” (KII3, researcher, Cape Town).*

The interpersonal challenges involve the safety of mobile teams being compromised, especially when some FSWs experience altercations with their clients in certain territories.

*“The challenge we face working with sex workers is that some of them do what they refer to as swimming, while one of them is busy with the client, then the others will crawl inside the room and steal the client’s wallet. One time it happened that a client came wanting to attack the sex workers and our team was there doing outreach, and the sex workers ran inside the mobile. We also work in Hillbrow and we do not have a safe space to park our mobile, so anything can happen, and we have to have pepper sprays for our team” (KII4, researcher, Johannesburg).*

The integration of FSWs into public health care facilities is another structural challenge because most public clinics and hospitals need sensitisation to provide services in a non-discriminatory manner. This training is crucial because these government facilities are ultimately expected to be the primary source of care and to take over from these organisations once their programmes come to an end.

*“To make this a sustainable project, the mobile unit is a five-year project and the focus is reintegrating sex workers back into public health care facilities. The mobile unit is about building that first level of trust with health care again. In Cape Town we know that there are four facilities that sex workers like to go to, but we want to identify other clinics and sensitise them and see how well that will work in getting sex workers to access public health care” (KII3, researcher, Cape Town).*

## Discussion

FSWs’ experiences documented in this study and other extensive research conducted in South Africa shows that health care providers in public health care facilities have proven ineffective in providing services to FSWs in a way that is beneficial to FSWs as patients [25–28]. As service providers, the health sector should be providing a service in a way that recognises and upholds the patient’s right to care. On the contrary, FSWs who open up regarding their sex worker identity are exposed to stigma and discrimination that strip them of their personal dignity. Research by Lafort and colleagues [28] has shown that 84% of FSWs in Durban received their last STI diagnosis at a public health facility. This could possibly mean that a large number of FSWs are exposed to unpleasant health services, compounding their state of vulnerability.

As shown in this study, there are several SRH interventions run by non-governmental health and advocacy organisations. These interventions are in the form of sex worker sensitive clinics and mobile outreach health facilities where FSWs access health care free from discrimination. This has resulted in the normalising of HIV testing among FSWs. Through peer support, these mobile units also provide care to brothel and hotel-based sex workers and deliver STI treatment kits and condoms, and currently deliver PrEP directly to FSWs [29, 30]. Furthermore, consumer research has shown that in order for a service to be impactful it is important to engage in the co-creation of services with consumers in ways that are collaborative to achieve well-being [31–33]. These health and advocacy organisations have achieved co-creation through the establishment and use of the peer education approach. Through this co-creation, FSWs are not simply recipients of services but have become part of service design and delivery, thus achieving greater impact.

In mainstream health care, patients, particularly those from marginalised groups have expressed feelings of being treated as less knowledgeable and experienced a sense of discomfort when engaging with their health care providers. This creates a lot of anxiety for patients during the consultation process [34, 35]. However, these health and advocacy organisations have followed a trust-building approach which encourages full disclosure and respectful engagement between FSWs and health care providers. This decreases disparities between patients and health care providers and creates an empowering environment whereby the patient takes ownership of their own health and well-being enforced by the positve interaction [36]. This approach can be effective for treatment initiation and adherence to ARVs and PrEP, which is important for HIV prevention. Through this approach it is possible for health care providers to confront issues of social justice whereby marginalised groups are helped to mitigate their vulnerability, and achieve a sense of agency and an empowered approach to their health.

## Conclusion

It is evident that the non-governmental health and advocacy organisations in this context have taken the initiative to provide health care access to FSWs through peer-led SRH care and psychosocial services.  These organisations have ensured that they provide services to FSWs in a way that fosters a trusting and respecful interaction between FSWs and health care providers. The power imbalances that characterise the health care provider and patient dynamics in most public health facilities have been replaced with a system where FSWs communicate freely with health care providers about their sex worker identity, as well as receiving a service tailored to the complex challenges they experience in sex work. However, it is important to note that these organisations are limited in their reach and are not available to sex workers operating outside of the metropolitan municipalities; thus, sex workers who operate in townships or rural settings may rely heavily on public clinics and hospitals enforcing a sense of powerlessness and lack of well-being among some members of this population, fostering health disparities due to the inaccessibility of supportive services. In its quest for health care reform, the South African health sector should engage with these organisations and aim to design government-led parallel services that have a wider reach, and with sensitised health care staff so as to gradually cater for key populations.

### Limitations

This analysis is subject to several limitations: The findings from this study cannot easily be generalised to represent the knowledge or practices of FSW populations, as they are drawn from qualitative research with a purposively recruited sample. During FGDs, some participants’ responses could have been influenced by what their peers were saying or the responses of facilitators, which could have led to bias responses. Furthermore, the study does not report on the health care challenges faced by other groups of sex workers such as males, men who have sex with men and transgender sex workers.

## Supplementary information

**Additional file 1.** Interview guide for key informant interviews.

**Additional file 2.** Interview guide for focus group discussions.

## Data Availability

The datasets used and/or analysed during the current study are available from the corresponding author on reasonable request.

## Abbreviations

ART: Antiretroviral treatment
ARV: Antiretroviral
FGDs: Focus group discussions
FSWs: Female sex workers
IBBS: Integrated biobehavioural survey
KIIs: Key informant interviews
PrEP: Pre-exposure prophylaxis
SRH: Sexual reproductive health
STI: Sexually transmitted infections
SWEAT: Sex Worker Advocacy Taskforce
THCA: TB/HIV Care Association
Wits RHI: Wits Reproductive Health and HIV Institute
