# Supplementary material for: The role of health and advocacy organisations in assisting female sex workers to gain access to health care in South Africa
Source: BMC Health Serv Res. 2019 Oct 24;19:746. doi: 10.1186/s12913-019-4552-9 (PMC6814112; doi:10.1186/s12913-019-4552-9)
Supplement: Supplementary file 1 — Additional file 1. Interview guide for key informant interviews. [file 12913_2019_4552_MOESM1_ESM.docx]

**Interview schedule 1. Formative assessment for inner city FSWs to establish the feasibility of conducting an integrated bio behavioral survey in the cities of Durban Johannesburg and Cape Town**

Key informant interview guide for FSWs coordinators, peers, researchers and stakeholders

To start our session, I would like for you to please introduce yourself, by telling us your name and you don’t need to tell us your real name, then your age, nationality and where do you stay, as well as your educational level and language.

1. What kind of services does your organization provide?
2. What is your role in the organization?
3. What kind of contact do you have with female sex workers?
4. How would you describe the female sex worker population?
5. Do you think that they are different subgroups of female sex workers in this area?
6. Can you describe the different places where they operate?
7. Can you give specific names and specific streets as well?
8. Do sex workers in this area operate through pimps or agents?
9. What could you say is the type that has a pimp?
10. Besides the streets and the brothels, are there any other ways that you know of that sex workers find clients?
11. Of all the places mentioned, approximately how many female sex workers do you know that come to each of those places, or that operate in those places?
12. Do you know about the mobility of the sex work population, do they like to move of in and out of town a lot?
13. Do you know of any sex workers that are under the age of 18?
14. Do you think these underage girls are being trafficked?
15. Is it a lot of these girls that you see that are under-age?
16. How do the underage girls start selling sex?
17. What challenges or successes has your organization experienced since you started working with the sex worker population?
18. How did your organization built trust with them?
19. Apart from Lifeline and your organization, are they any other organizations that focus on this population?
20. Is there a substantial population of male sex workers?
21. I just want to ask you about potential sampling, for when we start the survey, we have two methods first one is RDS where we give participants invitation cards to hand out to their friends who are sex workers to also come and participate. There is another strategy where we actually go to the venues where sex workers operate, and we recruit them to come and then get tested and go through survey questionnaire, then get reimbursed. Do you think that will work?
22. Which method would bring in more sex workers? The first or the second?
23. One other way of trying to get a population estimate is to organize a unique event where we give out unique objects to each sex worker. Later when they participate in the survey, we will ask them if they have received this unique object. What object do you think we should get? We are trying to get ideas of what the best unique object would be, because first, we don’t want it to be too cheap, but we also don’t want it to be so expensive that some may pretend to be sex workers, so that they can get it.
24. What languages do most female sex workers not feel comfortable speaking?
25. As part of the health study we will test for HIV and syphilis, and this will involve a rapid test as well as taking blood to the lab. Do you think sex workers will be comfortable with that?
26. We won’t be taking any names during the survey, and we do not want sex workers to give us their real names, but we will want to make sure that people do not come in twice, and to prevent this we will have a fingerprint scanner, but the scanner does not save the fingerprint, it just takes the image and then creates a computer generated code that is unique to that person. Do you think that sex workers will be comfortable with that?
27. We are also looking for a study site that will be convenient and safe for sex workers to access, which area do you think will be good for us to locate our office?
28. Do you know of any locations that we should avoid?
29. What do you think would be the perfect reimbursement?
30. Some of the questionnaire in the survey will be very personal and we will be asking sex workers about their sexual partnerships and sexual history, do you think they will be comfortable in answering them honestly?
31. A MacBook will be used to enter participants’ responses, do you think sex workers will be comfortable with the use of computers during the survey?
32. The whole survey will take between 2 and 3 hours, that includes the interview, the pre and post counselling. Do you think they will be willing to commit to this time?
33. Do you pay them each time when you go out with those mobile visits?
34. Do you think that various subgroups of female sex workers from various communities would be able to get recruited into our study?
35. Do you have any questions for us?

Thank you!
