# Supplementary material for: The role of health and advocacy organisations in assisting female sex workers to gain access to health care in South Africa
Source: BMC Health Serv Res. 2019 Oct 24;19:746. doi: 10.1186/s12913-019-4552-9 (PMC6814112; doi:10.1186/s12913-019-4552-9)
Supplement: Supplementary file 2 — Additional file 2. Interview guide for focus group discussions. [file 12913_2019_4552_MOESM2_ESM.docx]

**Interview schedule 2. Formative assessment for inner city FSWs to establish the feasibility of conducting an integrated bio behavioral survey**

**Focus Group guide**

To start our session, I would like for to go around the group and can everyone please introduce themselves, by telling us your name, and you don’t need to tell us your real name, then your age, nationality and where do you stay, as well as your educational level and language.

**Who are FSWs?**

1. Is there anyone who has questions before we start?
2. We are going to talk about women who exchange sex for money, female sex workers: and can you describe the different types or groups of female sex workers in the community. How can you describe women who are doing sex work, what kind of people are they? Where do they work? Which age do they start doing this work? and which race groups do the women who do sex work in this city identify with?

1. **Where do they work?**
2. Would you say that age pushes sex workers into working in a specific place?
3. Does income influences sex workers into working in specific areas?
4. Can we say ethnicity motivates sex workers to work in a specific area?
5. Does education influence where the ladies choose to do sex work?
6. Do different female sex workers look for different clients, are they picky and choosy regarding the kinds of clients they service?
7. We want to talk about the places where sex work occurs and please just name the place then we will write the areas down. Which streets are popular for those who are working in outdoors?
8. What is the estimated number of sex workers who are working in these different places?
9. When do the places and venues mentioned get busy?
10. What can you say is the number of sex workers in this city?
11. Are they any sex workers who are working for pimps?
12. For those who are working for pimps, how do they treat them?
13. Are there women who are sex workers who are in stable partnerships (marriage, live in partner) whose partners don’t know that they are doing sex work?
14. Are there any sex workers who are advertising themselves on the internet and newspapers? (Please provide us with the names of those news-papers and websites)
15. Do female sex workers spend time together when they are not working to just have some fun?
16. How do sex workers refer to their clients?
17. Do you have different names for different clients?
18. Where else do sex workers working in this city come from?
19. Are they any male sex workers?
20. Where do they work? (Can we provide an estimate of how many male sex workers are they?)
21. Are there certain times of the year or some months whereby there are a lot of clients that are buying sex?

**Health care**

1. How do FSWs access health care?
2. What challenges do they experience in accessing health care?
3. Which organizations provide health care to FSWs?
4. Which other support organizations provide health care to sex workers?
5. What kinds of services do these organizations provide?
6. Which clinics or hospitals do sex workers feel comfortable going to, in the case that they are being referred?

**Feasibility of conducting an IBBS study**

1. We want to design a program that is going to help us understand the HIV prevalence in this city, so we want to have a survey that will help us count how many female sex workers that are in this city. Basically, we are going to have an office, and when a female sex worker comes into our office we are going to do HIV and syphilis testing. So we are going to have pre and post counselling, and we are going to have an interview. The whole process can take up to 2 to 3 hours each person. We want to know if sex workers will be willing to come into the office for 2 to 3 hours, is this time too long, or too little or they might not want to come at all?
2. We will do HIV testing and syphilis and we will draw the blood from the arm, will some sex workers allow us to do that or will they refuse to get tested?
3. We will not require people to give us their names during the survey, but we need to have a way to know whether this person got tested, and for the test results to be given to the right person. What we want is to have a fingerprint scanner and when you put your finger there, it does not take your fingerprint, but it transforms the fingerprint into a unique number. This unique identifier will prevent people from participating more than once into study, we want to count each person once. Do you think using the fingerprint scanner will make people scared to participate? Will they think that we are taking their fingerprints?
4. We are going to have a survey interview, and the sex workers will have to respond to questions from computer. Do you think they will have a problem with the use of computers?
5. We want to have a study office, which place do you think is safe and convenient to locate our offices? We want to choose an area that is central and not too far for sex workers to travel and where they will feel comfortable? (Can we have at least two options? How much is the bus fare from local and far places?)
6. Which days of the week can we open this office and the time?
7. Which languages can we use in the offices?
8. Can most female sex workers read and speak English?
9. As we have said that we are going to do a survey and we want 400 sex workers and that is a huge number, so we want to find a way of getting many people, maybe about 200 in a period of 6 months. We have two methods we would like to use to recruit participants, and the first one is called RDS, whereby the first sex worker to come and get tested into our offices will be give 3 invitation cards or coupons that she will give other sex workers, and if all those 3 people invited come, participate and get tested, that person will be given an incentive, and so on. The second option is to go into places where the ladies are working whereby we will call them to come do the survey and give them an incentive. For the first option you will get two incentives, one for coming and the second one for recruiting 3 people, however, with the second option you will get one incentive for getting yourself tested. Which option do you think it can help us recruit as many sex workers as possible?
10. We also have a sample of the invitation card/coupon and you can check it and suggest what can be changed there, in terms of size, what should be written on the card?
11. How long do you think it can take one person to come and get tested after receiving the invitation card?
12. What can prevent people from not coming?
13. We want you to suggestions the people we can start giving the invitation cards to first, because it should be a popular person, who is known by many people?
14. Do we have any other people who are supposed to be in this group but aren’t here now?
15. Before we start the survey, we want to do something, called a unique event, and we want to have as many female sex workers as possible. So, we would like suggestions on what can we do and what can the event incorporate, so that we can get as many ladies as possible, can it be a party, picnic or braai? We want people to know us, so we want to introduce ourselves to the ladies.
16. We will give people a unique object at the event, to know how many people came, so what do you suggest can be the unique object? and we don’t want to give away expensive things because people might be tempted to sell it. We were thinking about giving out the rubber bangles, but you can give us any other suggestions?
17. Which days of the week and time should we do the event, so that people will not have excuses for not attending?
18. Which incentives do you think we can give you guys for participating in the survey?
19. Which incentives can we give to those who managed to recruit 3 people by giving them the invitation cards?
20. Do you have any questions you may like to ask?

Thank you!
